# Research progress of cerebroplacental ratio in obstetric complications

**DOI:** 10.3389/fmed.2026.1876901

**Published:** 2026-08-27

**Authors:** Xinran Bi, Ruotong Qi, Haiying Chen

**Affiliations:** Department of Obstetrics, The First Hospital of China Medical University, Shenyang, China

**Keywords:** brain-sparing effect, cerebroplacental ratio, Doppler ultrasound, fetal hemodynamics, obstetric complications

## Abstract

Cerebroplacental ratio (CPR), defined as fetal middle cerebral artery pulsatility index (MCA-PI)/umbilical artery pulsatility index (UA-PI), quantifies intrauterine hemodynamic redistribution driven by the fetal brain-sparing response to placental insufficiency. With the advancement of ultrasonic technology and in-depth clinical research, CPR has been widely applied in early screening, condition assessment, prognosis evaluation and intervention guidance of various obstetric complications, especially showing unique clinical value in high-risk pregnancies such as fetal growth restriction, preeclampsia and hyperglycemia in pregnancy. For example, in fetal growth restriction (FGR), low CPR yields 78.5% sensitivity and 82.1% specificity for predicting intrapartum fetal distress, with an adjusted OR of 6.82 for composite adverse perinatal events. This review aims to collect literature about the roles of CPR in pregnancy complications. We consider all relevant articles in English from 1987 to 2026 through mainstream academic databases, including PubMed, Web of Science, CNKI and Wanfang Data. We systematically describe the physiological basis and measurement criteria of CPR, elaborates on its research progress in common obstetric complications, analyzes the controversies and deficiencies of current studies, and prospects its future clinical application, so as to provide theoretical and practical support for the precise management of high-risk obstetric pregnancies.

## 1 Introduction

Obstetric complications are the leading cause of adverse pregnancy outcomes in pregnant women, as well as elevated perinatal morbidity and mortality rates. Although traditional antenatal monitoring methods, including fetal heart rate monitoring, amniotic fluid index and fetal biophysical profile scoring, can preliminarily evaluate fetal intrauterine wellbeing, they exhibit inherent limitations in the early identification of subclinical placental dysfunction and the prediction of adverse pregnancy outcomes, failing to meet the clinical demands of precision obstetrics (1).

Since first reported by Arbeille et al. (2), CPR has gradually become a research hotspot in the field of obstetrics. By quantifying the hemodynamic parameter ratio between the fetal middle cerebral artery and the umbilical artery, CPR indirectly reflects the balance between placental circulatory resistance and fetal cerebral perfusion, enabling the early detection of the fetal compensatory response to adverse intrauterine conditions such as hypoxia and placental insufficiency, namely the “brain-sparing effect.” When placental function is impaired, elevated placental circulatory resistance leads to increased umbilical artery flow resistance. To maintain the blood and oxygen supply to vital organs including the brain and heart, the fetus compensates by dilating intracerebral blood vessels and reducing cerebral vascular resistance, together with constricting peripheral vessels and decreasing perfusion of non-vital organs, resulting in hemodynamic redistribution. This process can be precisely reflected by abnormal changes in CPR. Compared with isolated umbilical artery or middle cerebral artery hemodynamic parameters, CPR can more comprehensively and sensitively indicate fetal intrauterine hemodynamic disturbance, providing a more reliable basis for the prediction and early intervention of obstetric complications (3, 4).

In recent years, scholars worldwide have conducted extensive clinical studies on the application of CPR in various obstetric complications. Relevant evidence has confirmed that CPR is of great value in the screening, condition evaluation and prognostic assessment of fetal growth restriction, preeclampsia and other related diseases. Nevertheless, several unresolved issues remain, including inconsistent cutoff values of CPR, incompletely clarified mechanisms underlying its role in different complications, and the lack of standardized combined detection protocols (5, 6). This review aims to collect literature about the roles of CPR in pregnancy complications. Two independent researchers searched PubMed, Web of Science, CNKI and Wanfang Data for articles with the following medical subject headings (MeSHs): “cerebroplacental ratio,” “fetal growth restriction,” “preeclampsia,” “hyperglycemia in pregnancy,” “twin pregnancy,” “preterm birth,” “intrauterine fetal demise,” “pregnant.” We consider all relevant articles in English from 1987 to 2026. We assessed study quality using NOS for observational studies and QUADAS-2 for diagnostic studies. Funnel plot analysis was conducted to evaluate potential publication bias in the enrolled literature. This review systematically summarizes the research progress of CPR in common obstetric complications, integrates the latest clinical evidence, and discusses its clinical application value, research prospects and existing limitations. It aims to provide evidence-based references for the precise management of high-risk pregnancy and promote the standardized clinical application of CPR.

## 2 Physiological basis and reference parameters of the cerebroplacental ratio

### 2.1 Physiological basis

In normal pregnancy, with advancing gestational age, the placenta gradually matures accompanied by proliferation and branching of the placental vascular bed, resulting in a progressive reduction in placental circulatory resistance. Correspondingly, UA-PI shows a continuous downward trend across gestation. As fetal cerebral development progresses, cerebral blood flow demand increases, the middle cerebral artery dilates, and cerebral vascular resistance gradually decreases, leading to a slow decline in MCA-PI with advancing gestational age. Nevertheless, MCA-PI remains consistently higher than UA-PI throughout pregnancy (7). Under such physiological conditions, CPR is maintained within a relatively stable normal range. Changes in CPR can reflect the balance between placental circulation and cerebral perfusion, indicating a favorable fetal intrauterine status.

When the fetus is exposed to adverse intrauterine conditions such as hypoxia and placental insufficiency, the brain-sparing effect is activated, which constitutes the core pathological mechanism underlying abnormal CPR. In this setting, increased placental circulatory resistance leads to an elevation in UA-PI; meanwhile, compensatory dilatation of fetal intracranial blood vessels results in a reduction in MCA-PI. The combined changes of the two indices ultimately cause a decrease in CPR. The magnitude of CPR reduction is positively correlated with the severity of fetal hypoxia. In mild hypoxia, CPR decreases slightly, and fetal cerebral blood supply can be temporarily preserved through hemodynamic redistribution. In severe hypoxia, a marked decline in CPR indicates insufficient fetal compensatory capacity, which may lead to brain injury, intrauterine distress, and even fetal demise. In addition, the normal range of CPR is also influenced by individual differences in fetal hemodynamics, gestational variation, and maternal physiological status, suggesting that clinical interpretation should be based on comprehensive clinical evaluation.

### 2.2 Reference parameters

#### 2.2.1 Normal reference ranges and cutoff values

The normal reference range of the CPR is affected by multiple factors, including gestational age, fetal gender, ethnicity, testing equipment and detection methods. At present, a unified international standard has not been established, and the definition of the cutoff value remains the most controversial issue. There are two main commonly used criteria for determining the clinical cutoff of CPR. One adopts the gestation-specific 5th percentile, whereby a CPR value below the 5th percentile of the normal population at the corresponding gestational age is defined as abnormal (8). The other applies a fixed cutoff value, with CPR < 1.0 and CPR < 1.08 being the most widely used thresholds (9). The 5th percentile value specific to gestational age is characterized by higher specificity and better conformity to the physiological variations of CPR across different gestational weeks. It is suitable for targeted screening in patients with fetal growth restriction and high-risk pregnancies, and can effectively reduce false-positive results (4). Fixed cutoff values (especially CPR < 1.08) exhibit higher sensitivity, facilitate standardized implementation, and are suitable for rapid screening and risk stratification in unselected late-third-trimester populations (4, 10). In addition, the normal range of CPR varies across gestational weeks. The MCA-PI reaches its peak at 28–32 weeks of gestation and declines thereafter; accordingly, CPR remains at a relatively high level during 28–32 weeks, followed by a slight decrease with advancing gestational age.

**FIGURE 1:**
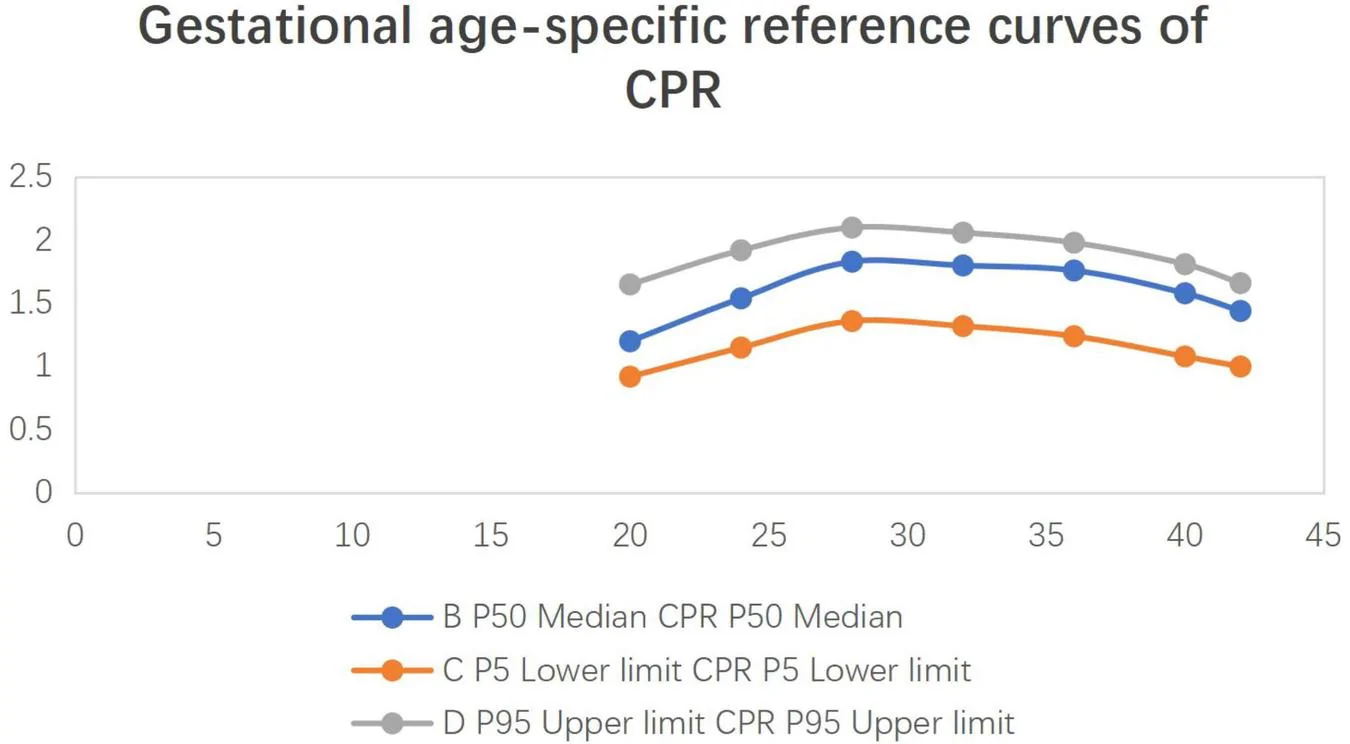
Gestational age-specific reference curves of CPR.

In recent years, several multicenter studies have attempted to establish gestation-specific reference ranges for CPR. For instance, a large-sample multicenter study constructed gestation-specific percentile reference curves for UA-PI, MCA-PI, and CPR across 20–42 weeks of gestation. The results demonstrated that UA-PI decreased continuously with advancing gestational age, whereas both MCA-PI and CPR presented an initial increase followed by a subsequent decline. At 40 weeks of gestation, the median values of UA-PI, MCA-PI and CPR were 0.87, 1.32, and 1.58, respectively. The relevant reference curve is presented in Figure 1. This standard is applicable to the standardized hemodynamic assessment and risk stratification of fetuses. Another study focusing on twin pregnancies found that the normal CPR reference range in twins was lower than that in singleton pregnancies, and distinct differences existed between monochorionic diamniotic (MCDA) and dichorionic diamniotic (DCDA) twins (11). Furthermore, racial disparities in CPR reference ranges have been well documented. A comparative study between Asian and European-American populations indicated that the mean fetal CPR value in Asian pregnant women was slightly lower than that in Western populations (12). Accordingly, the gestation-specific reference range should be prioritized in clinical practice. Comprehensive evaluation of abnormal CPR should incorporate fetal individual characteristics, ethnic background and diagnostic equipment, so as to avoid misdiagnosis or missed diagnosis caused by the application of a single fixed cutoff value.

#### 2.2.2 Factors affecting the detection results

The detection results of CPR are influenced by multiple confounding factors, which should be fully emphasized in clinical practice to guarantee the accuracy and reliability of measurements: ➀Gestational age: As mentioned above, gestational age is the predominant factor affecting CPR. The normal reference range of CPR varies significantly across different gestational weeks. Accurate gestational age confirmation is essential before examination to prevent misinterpretation caused by gestational age miscalculation (13). ➁Fetal status: Fetal movement, breathing activity, and sleep–wake cycle can all affect Doppler flow spectra. Frequent fetal movement or the awake state may destabilize blood flow signals and induce errors in PI measurement. It is recommended to perform ultrasound examination during fetal quiescence; if fetal movement is persistent, the scanning time should be appropriately prolonged or the examination temporarily suspended. ➂Maternal factors: Maternal posture (supine position may cause uterine compression of the abdominal aorta and alter fetal hemodynamics), blood pressure (maternal hypertension or hypotension), blood glucose (poor glycemic control in patients with gestational diabetes mellitus), and emotional state (excessive stress and anxiety may affect maternal circulation and indirectly disturb fetal hemodynamics) can indirectly interfere with fetal hemodynamic parameters and further influence CPR results. Pregnant women are advised to maintain a relaxed mood and adopt a comfortable position during detection (14, 15). ➃Examination technique: Operational factors including probe angle, selection of flow spectrum waveform, and measurement frequency substantially affect the precision of PI detection. Examinations should be performed by experienced sonographers in strict accordance with standardized protocols. Continuous and stable Doppler spectra should be acquired, and measurements should be repeated at least three times to calculate the mean value so as to reduce observational bias (16). ➄Fetal abnormalities: Fetal structural malformations (such as central nervous system anomalies and cardiovascular malformations) and chromosomal abnormalities may directly alter cerebral perfusion or placental circulation, leading to abnormal CPR values. Comprehensive assessment combining other auxiliary examinations (e.g., fetal echocardiography and non-invasive prenatal testing) is therefore required (17). ➅Placental factors: Placental abnormalities such as placenta previa, placental abruption, and abnormal placental morphology can change placental vascular resistance and subsequently affect the final CPR measurement.

## 3 Application of the cerebroplacental ratio in common obstetric complications

### 3.1 Fetal growth restriction

Fetal growth restriction (FGR) is defined as a condition in which the fetus fails to reach its inherent growth potential due to pathological factors involving the mother, fetus, placenta and umbilical cord. In most cases, the estimated fetal weight (EFW) or abdominal circumference is below the 10th percentile for gestational age. Severe FGR is defined as a value below the 3rd percentile or accompanied by abnormal Doppler flow parameters, which is associated with a markedly elevated risk of adverse pregnancy outcomes and constitutes one of the most common high-risk obstetric complications. The incidence of FGR is approximately 5–10%. It not only increases the risks of perinatal asphyxia, hypoxic-ischemic encephalopathy and other neonatal morbidities, but also raises neonatal mortality. Moreover, surviving offspring have a significantly higher long-term risk of developing metabolic disorders such as hypertension and diabetes, as well as cardiovascular diseases in adulthood.

The development of FGR is closely linked to a variety of fetal and maternal pathological factors. In terms of fetal factors, chromosomal abnormalities, congenital malformations, intrauterine infection, and complications of multiple pregnancy can directly impair fetal growth potential. From the maternal perspective, gestational vascular diseases, chronic underlying illnesses, metabolic disorders, nutritional imbalance, and uterine structural abnormalities may result in impaired uteroplacental perfusion. This impedes the transplacental transport of oxygen and nutrients, thereby collectively contributing to the onset of fetal growth restriction.

CPR is of pivotal clinical value for pathological FGR caused by placental insufficiency and uteroplacental circulatory disturbance, and it is particularly applicable to late-onset FGR. CPR can effectively identify occult FGR with normal umbilical artery Doppler parameters, compensating for the limitations of single-vessel Doppler assessment. In early-onset FGR, CPR enables multi-parameter combined evaluation of the severity of placental dysfunction and the level of fetal hypoxic compensation. Meanwhile, CPR can differentiate physiological small-for-gestational-age fetuses from true FGR, and distinguish placental-origin FGR from non-placental fetal growth abnormalities attributable to chromosomal anomalies or structural malformations. Furthermore, it allows severity stratification and dynamic progression assessment of placental-origin FGR, independently predicts adverse perinatal outcomes, and provides an important evidence-based reference for clinical monitoring and optimal timing of delivery.

Early identification and timely intervention of FGR are critical to improving perinatal prognosis. As a sensitive indicator reflecting fetal hemodynamic disturbance, CPR possesses important clinical value in the diagnosis, severity evaluation and prognostic assessment of FGR.

#### 3.1.1 Diagnostic correlation between CPR and FGR

Impaired placental function leads to intrauterine fetal hypoxia and insufficient nutrient supply, thereby resulting in FGR. CPR enables early detection of hemodynamic alterations underlying this pathological process. A number of studies have confirmed that the abnormal rate of CPR in FGR fetuses is significantly higher than that in normal fetuses (18, 19), and abnormal CPR usually emerges earlier than conventional monitoring markers such as abnormal fetal heart rate patterns and oligohydramnios. A study conducted by Leavitt et al. demonstrated that abnormal CPR may be present in suspected FGR even when umbilical artery Doppler parameters remain normal. This indicates that CPR can screen for occult FGR with normal umbilical artery blood flow, overcoming the limitations of isolated umbilical artery assessment (20). Although such occult FGR fetuses present no obvious umbilical artery Doppler abnormalities, they already have subclinical placental dysfunction. CPR examination allows early identification and provides a valuable time window for clinical intervention. When umbilical artery Doppler abnormalities occur, abnormal CPR is closely correlated with reduced fetal growth rate. By contrast, fetuses with abnormal umbilical artery flow but normal CPR exhibit growth rates similar to those with normal umbilical artery parameters, suggesting that CPR serves as a reliable marker for determining true fetal growth restriction (21). Given the pivotal role of CPR in FGR diagnosis, the 2017 Delphi consensus incorporated CPR below the gestation-specific 5th percentile into the diagnostic reference indicators for FGR. Combined with abnormal umbilical artery Doppler and reduced amniotic fluid index, CPR can improve the overall diagnostic accuracy of FGR (8). Notably, small for gestational age (SGA) and FGR are not equivalent concepts. Approximately 50–70% of SGA cases represent constitutionally small stature determined by genetic factors, without placental dysfunction or increased risk of adverse perinatal outcomes. In contrast, FGR is predominantly caused by placental insufficiency and is strongly associated with adverse pregnancy outcomes. CPR can effectively distinguish SGA from FGR: the incidence of abnormal CPR is markedly higher in FGR fetuses than in SGA counterparts. Moreover, SGA fetuses with abnormal CPR carry a significantly elevated risk of adverse pregnancy outcomes, whereas those with normal CPR generally have a favorable prognosis (22, 23). In addition, CPR facilitates the differential diagnosis of FGR from other conditions causing aberrant fetal growth, such as chromosomal abnormalities and structural malformations. For instance, although fetuses with chromosomal anomalies also show a high rate of abnormal CPR, such cases are usually accompanied by additional abnormal ultrasonic soft markers. In comparison, CPR abnormalities in FGR are primarily attributed to underlying placental dysfunction (24).

#### 3.1.2 Correlation of CPR with FGR severity

Abnormal CPR can be used as an objective basis for severity stratification of FGR. In normal pregnancy, fetal CPR generally remains above 1.0. In FGR, however, reduced placental perfusion, elevated umbilical artery resistance, and compensatory cerebral vasodilation commonly lead to a decline in CPR. A CPR value below the diagnostic cutoff, typically set at less than 1.0 or below the 10th percentile, suggests underlying fetal hemodynamic disturbance. A substantial or progressive reduction in CPR indicates worsening placental insufficiency and near depletion of fetal compensatory reserve, corresponding to moderate and severe stages of FGR (25). Accumulated evidence has shown that CPR levels correlate well with the severity of placental pathological lesions. A lower CPR is accompanied by more severe inadequate maternal placental vascular perfusion, underdevelopment of distal villi, and abnormal vascular muscularization. Such pathological changes further confirm that CPR can reliably reflect the intrinsic severity of FGR (19, 26).

In terms of clinical phenotypic stratification, CPR exhibits distinct implications for severity assessment between early-onset and late-onset FGR (10). Early-onset FGR, defined as onset before 32–34 weeks of gestation, is clinically more critical and frequently complicated by marked placental insufficiency. In such cases, CPR abnormalities occur earlier with a more pronounced reduction, often accompanied by absent or reversed end-diastolic flow (AEDF/REDF) in the umbilical artery and abnormal ductus venosus waveforms. These changes indicate a substantially elevated risk of severe fetal hypoxia and acidosis (27, 28). In contrast, late-onset FGR is more commonly characterized by an isolated reduction in CPR, while umbilical artery Doppler parameters may remain within the normal range. This pattern suggests predominantly mild-to-moderate fetal hypoxia with a relatively stable clinical condition. Nevertheless, a decreased CPR still effectively predicts intrapartum fetal distress and short-term adverse neonatal outcomes (29). Therefore, CPR not only differentiates pathological FGR from constitutional small-for-gestational-age fetuses, but also enables internal severity stratification within FGR, providing a reliable reference for clinical evaluation of fetal intrauterine wellbeing.

Several systematic reviews and meta-analyses further validate the value of CPR in assessing the severity of FGR (4, 10, 30). Abnormal CPR is significantly associated with an increased incidence of delivery by cesarean section, neonatal admission to the neonatal intensive care unit, low Apgar scores, preterm birth, and adverse perinatal events. Moreover, its predictive performance is generally superior to that of isolated umbilical artery or middle cerebral artery Doppler parameters. Dynamic monitoring of serial CPR trends is also of great clinical importance. A progressive decline in CPR among FGR fetuses indicates continuous disease deterioration and gradual depletion of fetal compensatory mechanisms, serving as an important clinical indicator for intensified surveillance and timely termination of pregnancy. In addition, combined application with other Doppler markers, such as ductus venosus flow and the umbilicocerebral ratio (UCR), can further improve the predictive accuracy for FGR severity and perinatal outcomes (31, 32).

#### 3.1.3 Prognostic value of CPR in FGR

CPR is not only applicable to the diagnosis and severity assessment of FGR, but also enables effective prediction of perinatal prognosis in FGR fetuses, providing evidence for clinical decision-making (10). Multiple studies have confirmed that abnormal CPR acts as an independent risk factor for adverse perinatal outcomes in FGR fetuses, including intrauterine fetal demise, fetal distress, emergency cesarean delivery, neonatal asphyxia, neonatal acidosis, and neonatal intensive care unit (NICU) admission (4, 30, 33). Nassr et al. analyzed relevant studies on CPR published between 1992 and 2014 and found that FGR fetuses with abnormal CPR (defined as CPR < 1.08, CPR < 1.05, or CPR below the gestation-specific 5th percentile) exhibited a 6–10-fold higher risk of emergency cesarean delivery due to fetal distress and a 5-fold higher risk of neonatal acidosis compared with those with normal CPR. Meanwhile, abnormal CPR demonstrated high sensitivity in predicting fetal distress, 5-min Apgar score < 7, NICU admission, and other neonatal complications, with corresponding sensitivity values of 78.5, 82.1, and 76.3%, respectively (30). Another prospective study enrolled 120 FGR fetuses with follow-up until delivery. The results showed that the incidence of adverse outcomes reached 78.3% in the abnormal CPR group, which was significantly higher than 21.7% in the normal CPR group (*P* < 0.001). Multivariate logistic regression analysis identified abnormal CPR as an independent predictor of adverse perinatal outcomes in FGR fetuses (OR = 6.82, 95%CI: 3.21–14.50, *P*< 0.001) (18). Furthermore, dynamic changes in CPR confer greater prognostic value for FGR fetuses. If CPR gradually rises and returns to the normal range after clinical intervention, it indicates improved fetal hemodynamic disturbance and relieved hypoxic status, associated with a favorable prognosis. By contrast, a continuous or further decline in CPR suggests disease progression and aggravated fetal hypoxia, warranting timely termination of pregnancy to reduce perinatal mortality (32). In addition, serial CPR trends can guide the optimal timing of clinical intervention for FGR. For preterm pregnancies with persistently abnormal CPR, expectant management may be adopted under close surveillance; further deterioration of CPR necessitates prompt delivery. For cases with restored normal CPR, gestational age can be appropriately prolonged to enhance fetal maturity (24, 34, 35).

### 3.2 Preeclampsia

Preeclampsia (PE) is a pregnancy-specific disorder with an incidence of 2–8%, typically manifesting after 20 weeks of gestation. It is clinically characterized by hypertension and proteinuria. In severe cases, it may progress to convulsions, coma, and multiple organ failure, ranking among the leading causes of maternal and perinatal mortality worldwide. Its core pathogenesis is initiated by abnormal invasion and differentiation of placental trophoblasts and impaired remodeling of uterine spiral arteries. This leads to superficial placental implantation and placental ischemia and hypoxia, which stimulate the massive release of anti-angiogenic factors, inflammatory mediators, and oxidative stress factors into the circulation. Consequently, placental circulatory resistance increases, aggravating placental hypoperfusion and hypoxia, and ultimately triggering systemic maternal endothelial injury and fetal hemodynamic disturbance. As a sensitive marker reflecting fetal hemodynamic status, CPR enables early detection of fetal blood flow redistribution resulting from preeclampsia-related placental insufficiency. Therefore, CPR holds important clinical value in the early screening, severity evaluation, fetal wellbeing monitoring, and prognostic assessment of preeclampsia (6).

#### 3.2.1 Diagnostic value of CPR in early screening of preeclampsia

Early screening for preeclampsia is crucial for optimizing maternal and fetal prognosis. Conventional screening indicators such as blood pressure and proteinuria usually present obvious abnormalities only after 20 weeks of gestation, limiting their utility for early prediction and timely intervention. Defective placental vascular remodeling constitutes the core pathological alteration of preeclampsia, occurring well in advance of clinical manifestations. Insufficient remodeling of spiral arteries can be detected as early as 12–14 weeks of gestation, leading to elevated placental circulatory resistance (36). Numerous studies have demonstrated that the rate of abnormal CPR is significantly higher in fetuses of women who subsequently develop preeclampsia than in those with normal pregnancies, and such CPR alterations emerge prior to the onset of clinical preeclampsia symptoms (3, 37). Regan et al. found that compared with women who had normal umbilical artery Doppler and normal CPR, individuals with abnormal CPR had a subsequent severe preeclampsia rate as high as 52.5%, which was markedly higher than 5.1% in the normal group and 15.4% in the group with abnormal umbilical artery flow but normal CPR (*P* < 0.01). After multivariate adjustment, abnormal CPR yielded an odds ratio of 4.14 (95%CI: 2.59–6.61) for predicting severe preeclampsia. Moreover, cases with abnormal CPR not only faced a greatly increased risk of severe preeclampsia but also exhibited an earlier gestational age at disease onset and earlier delivery, indicating that abnormal CPR serves as a valuable predictor for both the occurrence and advanced onset of severe preeclampsia. Edgar Hernandez-Andrade et al. analyzed 2986 singleton pregnancies at 20–24 weeks of gestation. The results showed that a CPR below the 5th percentile significantly predicted subsequent preeclampsia and fetal growth restriction. After adjusting for confounding factors, women with abnormal CPR had a 5.8-fold higher risk of developing preeclampsia than those with normal CPR (OR = 5.8, 95%CI: 2.6–13.0, *P* < 0.001). Additionally, mid-trimester CPR effectively predicted late-onset fetal growth restriction and low birth weight, suggesting that second-trimester CPR is a valuable biomarker for the early prediction of adverse pregnancy outcomes (38). Furthermore, the combined application of CPR with other early screening markers can further improve the overall screening efficiency for preeclampsia.

#### 3.2.2 Correlation between CPR and the severity of preeclampsia

The severity of preeclampsia is closely correlated with the degree of placental ischemia and hypoxia. Since the magnitude of CPR abnormalities can reflect the extent of placental insufficiency and fetal hypoxia, CPR is applicable to the severity assessment of preeclampsia. Patients diagnosed with severe preeclampsia suffer from substantial placental dysfunction and markedly elevated placental circulatory resistance, which aggravates fetal hypoxia and triggers an obvious cerebral protective effect, leading to a significant reduction in CPR (37, 39). A prospective cross-sectional study conducted in Uganda enrolled 128 pregnant women with preeclampsia, among whom 52.3% presented an abnormal CPR (CPR ≤ 1.0). The abnormal CPR rate was markedly higher in women with severe preeclampsia at 86.7%, with a statistically significant difference (*P* < 0.001). Multivariate analysis identified severe preeclampsia (adjusted prevalence ratio, APR = 5.0, 95%CI: 1.28–29.14) and eclampsia (APR = 5.27, 95%CI: 1.11–34.27) as independent risk factors for abnormal CPR. Differences in abnormal CPR rates also exist between early-onset and late-onset preeclampsia. Fetuses of women with early-onset preeclampsia demonstrate a significantly higher prevalence of abnormal CPR, which is attributed to more severe impairment of placental vascular remodeling and more profound placental ischemia and hypoxia in early-onset cases (40). In addition, abnormal CPR is associated with maternal complications in preeclampsia. Pregnant women with preeclampsia and abnormal CPR carry a remarkably higher risk of placental abruption, fetal growth restriction and preterm birth compared to those with normal CPR (3).

#### 3.2.3 CPR for monitoring fetal intrauterine status in preeclampsia

Pregnant women with preeclampsia suffer from impaired placental function, leading to a markedly elevated risk of fetal intrauterine hypoxia. Accordingly, monitoring fetal intrauterine condition is central to the clinical management of preeclampsia. Although conventional fetal monitoring methods such as fetal heart rate monitoring and amniotic fluid index can preliminarily evaluate fetal wellbeing, they exhibit relatively low sensitivity and limited capacity to detect early intrauterine hypoxia. In contrast, CPR can real-time reflect fetal hemodynamic disturbances and capture early warning signals of intrauterine hypoxia, thereby providing a more reliable basis for fetal surveillance (9). Numerous studies have confirmed that abnormal CPR in fetuses of preeclamptic women is closely associated with adverse outcomes including fetal distress and neonatal asphyxia (41). Furthermore, abnormal CPR is correlated with the long-term prognosis of preeclamptic fetuses. Those with altered CPR carry a significantly higher risk of neurodevelopmental delay and hypoxic-ischemic encephalopathy after birth compared with fetuses with normal CPR (42). Serial dynamic monitoring of CPR is of greater value in evaluating the intrauterine status of fetuses complicated by preeclampsia. Persistently normal CPR indicates stable fetal hemodynamics and satisfactory intrauterine condition, allowing expectant management under close surveillance. A gradual decline or emergence of abnormal CPR suggests progressive aggravation of fetal hypoxia, requiring intensified monitoring and timely termination of pregnancy when necessary to reduce perinatal mortality (39). Meanwhile, dynamic changes in CPR can also guide the optimal timing of delivery in preeclampsia.

### 3.3 Hyperglycemia in pregnancy

Hyperglycemia in pregnancy (HIP) is divided into three subtypes: pregestational diabetes mellitus in pregnancy (PGDM), prediabetes complicating pregnancy, and gestational diabetes mellitus (GDM). GDM is diagnosed based on prior diabetic history or early-onset hyperglycemia. GDM is screened via 75g OGTT at 24–28 weeks gestation, diagnosed once any glucose level exceeds the international cutoff values. Persistent glucose metabolic disturbance induces maternal vascular endothelial damage, placental oxidative stress and microcirculatory dysfunction, which subsequently lead to aberrant placental vascular remodeling, abnormal villous development and impaired uteroplacental perfusion. A sustained hyperglycemic intrauterine milieu can provoke chronic subclinical fetal hypoxia and initiate compensatory regulatory responses in the fetal cerebral circulation, resulting in reduced middle cerebral artery resistance and a declined CPR.

Women with poorly controlled hyperglycemia in pregnancy (HIP) exhibit a significantly higher incidence of abnormal fetal CPR, and such hemodynamic alterations can occur independently of fetal growth restriction. Fetuses exposed to hyperglycemia, insulin resistance and gestational metabolic disorders are more prone to reduced CPR compared with those under optimal glycemic control, indicating that an adverse intrauterine metabolic environment can directly disrupt the hemodynamic balance between cerebral and umbilical circulation. In addition, hyperglycemia in pregnancy is frequently complicated by macrosomia, polyhydramnios, and placental insufficiency. These comorbidities further increase fetal circulatory burden and exacerbate hypoxic status, thereby aggravating CPR abnormalities. Accordingly, CPR can objectively reflect the intrauterine compensatory reserve and hypoxic severity of fetuses affected by HIP, serving as a valuable ultrasonographic reference for fetal surveillance, disease severity assessment, and clinical intervention decision-making in such high-risk pregnancies.

#### 3.3.1 Features of CPR alterations in HIP fetuses

The alterations in the CPR of fetuses exposed to hyperglycemia in pregnancy (HIP) are complex and closely associated with glycemic control status and fetal growth patterns (43). Fetuses of mothers with pregestational diabetes mellitus (PGDM) exhibit characteristic dynamic changes in CPR. These alterations are jointly driven by placental vascular endothelial injury induced by prolonged hyperglycemia, fetal hyperinsulinemia, and dysregulated hemodynamic modulation. Compared with fetuses of GDM and normal pregnancies, PGDM fetuses develop cerebroplacental hemodynamic imbalance at an earlier gestational age. Specifically, CPR presents a mild compensatory elevation in the second trimester; in the third trimester, with progressive increases in umbilical artery resistance and declining compensatory capacity of the middle cerebral artery, CPR decreases progressively. Compared with normal fetuses, CPR changes in GDM fetuses manifest two distinct trends. First, poorly controlled GDM complicated with macrosomia may lead to reduced CPR due to elevated placental circulatory resistance and relative insufficient cerebral perfusion resulting from excessive fetal size (44). Second, GDM fetuses complicated by fetal growth restriction—mostly attributed to inadequate nutrient supply caused by placental vasculopathy—exhibit a typical cerebral protective response accompanied by a marked reduction in CPR (45). In contrast, GDM fetuses with optimal glycemic control maintain normal placental function and stable fetal hemodynamics, with CPR generally remaining within the normal reference range (44). A cross-sectional study conducted by Mohamed Waly et al. from the Department of Obstetrics and Gynecology, Cairo University School of Medicine enrolled 260 term pregnant women with GDM, who were divided into an insulin treatment group and a metformin treatment group. No significant intergroup difference in CPR values was observed. Among GDM fetuses with abnormal CPR (below the 10th percentile for gestational age), the incidence of adverse neonatal outcomes—including neonatal hypoglycemia, umbilical arterial pH < 7.25, 1- or 5-min Apgar score < 7, and neonatal intensive care unit admission longer than 24 h—reached 42.3%, which was significantly higher than 10.5% in the normal CPR group (46). In addition, CPR alterations in GDM fetuses are correlated with gestational age (47). Before 32 weeks of gestation, CPR levels show no significant difference between GDM and normal fetuses. After 32 weeks, CPR gradually declines in poorly controlled GDM fetuses, and the discrepancy versus normal fetuses becomes progressively evident. This may be explained by progressive placental maturation in late gestation, aggravated GDM-related placental vasculopathy, elevated placental circulatory resistance, and enhanced fetal cerebral protective response, ultimately resulting in a reduction in CPR. Furthermore, CPR variations in GDM fetuses are related to fetal sex. The rate of abnormal CPR is slightly higher in male than in female fetuses, with a statistically significant difference (*P*< 0.05). This phenomenon may be related to the higher susceptibility of male fetuses to adverse intrauterine environments (43).

#### 3.3.2 Correlation of CPR with adverse pregnancy outcomes complicating HIP

Abnormal CPR can effectively predict adverse perinatal outcomes in fetuses of pregnancies complicated by HIP, providing a reference for clinical management (48), with a stronger risk association observed in PGDM than in GDM. Sangeereni and colleagues conducted a prospective study enrolling 100 pregnant women with diabetes in pregnancy, including 42 cases of PGDM and 58 cases of GDM, to analyze the association between CPR and perinatal outcomes. The results showed that the mean CPR in the PGDM group was 1.21 ± 0.32, significantly lower than 1.48 ± 0.41 in the GDM group (*P* < 0.01). The proportion of abnormal CPR (CPR <1.3) in the PGDM group reached 38.1%. A reduced CPR was identified as an independent risk factor for adverse neonatal outcomes, with an OR of 4.23 (95%CI: 1.89–9.45) for predicting preterm birth, neonatal hypoglycemia, and NICU admission. The study confirmed more severe cerebroplacental hemodynamic imbalance in PGDM fetuses, and verified that CPR serves as a reliable ultrasonic indicator for predicting elevated perinatal risks in diabetic pregnancy (49). Multiple studies have demonstrated that abnormal CPR in GDM fetuses is closely correlated with adverse outcomes such as fetal distress, neonatal asphyxia, neonatal hypoglycemia, and NICU admission. Research by Hackmon et al. from Tel Aviv Medical Center revealed that the incidence of CPR below the 10th percentile was significantly elevated among women with GDM, particularly in those requiring insulin therapy. A low CPR was an independent risk factor for adverse perinatal outcomes and was significantly associated with composite adverse perinatal outcomes (OR = 2.93, 95%CI: 1.95–4.40, *P* < 0.001). It was also closely related to fetal distress, neonatal NICU admission, preterm birth, and low birth weight, indicating that reduced CPR is a valid ultrasonic marker for evaluating abnormal placental perfusion and increased perinatal risk in GDM fetuses. Relevant studies have shown that when CPR is below 1.0 in GDM fetuses, the incidence of adverse perinatal outcomes increases significantly, with a statistically significant difference (*P* < 0.001) (43). Notably, the predictive value of CPR in GDM has certain limitations. For instance, obstetric research from Cairo University School of Medicine reported that CPR exhibits low sensitivity (13.8%) but high specificity (96.1%) in predicting adverse outcomes in GDM fetuses, with an overall accuracy of 86.9%. This suggests that CPR should not be used alone as a screening tool for adverse outcomes in GDM fetuses and should be interpreted comprehensively in combination with other parameters. Combined application of CPR with fetal growth indices and amniotic fluid index can further improve the predictive efficiency for adverse perinatal outcomes in GDM fetuses (50).

#### 3.3.3 CPR and clinical management of hyperglycemia in pregnancy

**FIGURE 2:**
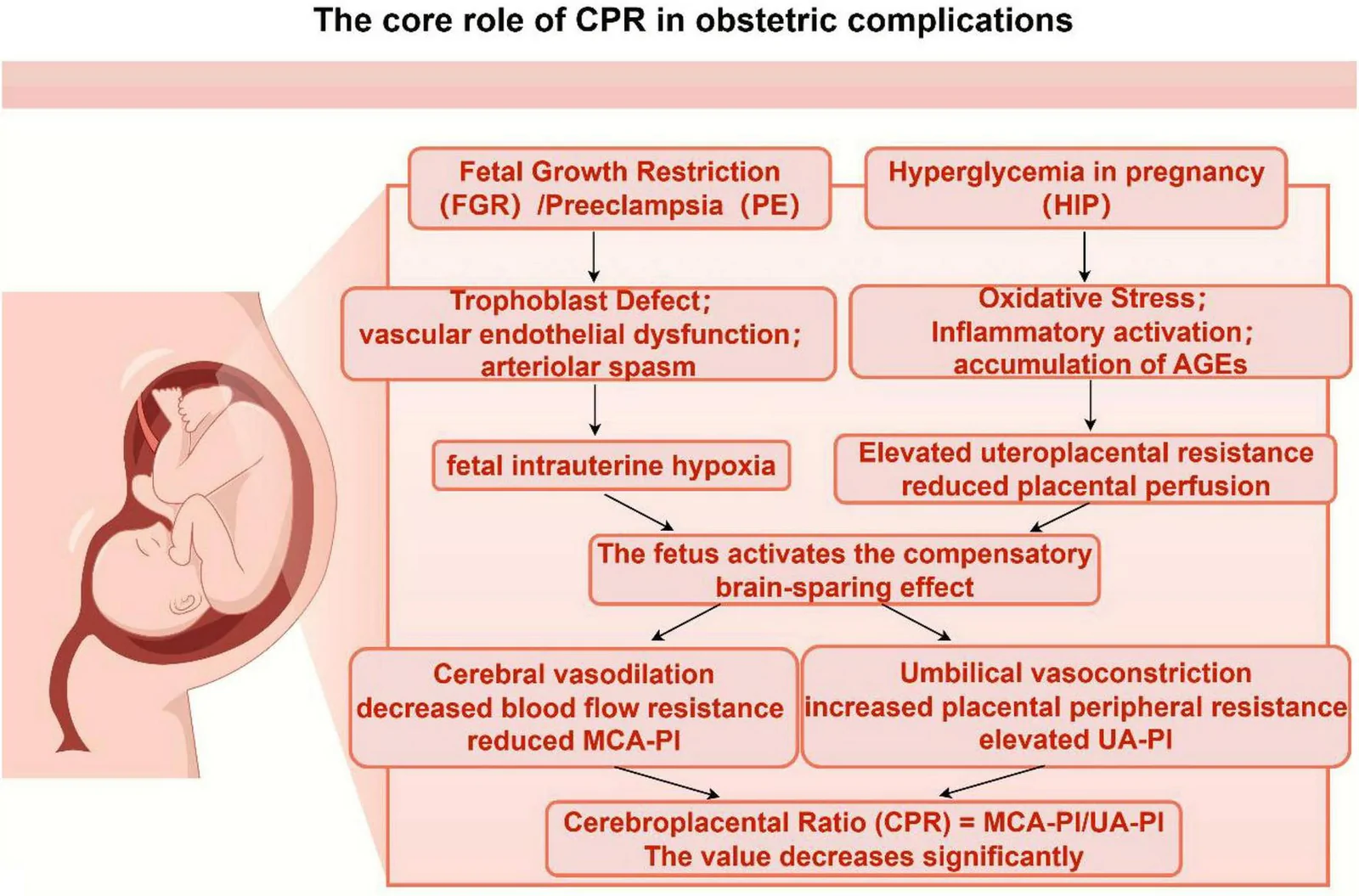
Mechanism underlying the role of CPR in obstetric complications. AGEs, Advanced glycation end products; MCA-PI, Middle cerebral artery pulsatility index; UA-PI, Umbilical artery pulsatility index.

CPR can serve as an important auxiliary indicator in the clinical management of pregnant women with HIP and guide glycemic regulation and obstetric intervention (48). For women with poorly controlled blood glucose, regular CPR measurement enables timely evaluation of fetal intrauterine status. A normal CPR indicates stable fetal hemodynamics, allowing continued optimization of glycemic control alongside close fetal surveillance. An abnormal CPR suggests a potential risk of fetal intrauterine hypoxia, necessitating prompt adjustment of the glycemic management regimen, intensified fetal monitoring, and timely termination of pregnancy when indicated. In addition, CPR is valuable for assessing the long-term prognosis of fetuses exposed to HIP. A small body of preliminary research tentatively indicates that infants of HIP pregnancies presenting low CPR could have a marginally increased theoretical risk of late-onset metabolic and cardiovascular comorbidities, which is postulated to arise from intrauterine hypoxic metabolic reprogramming. Notwithstanding this preliminary signal, current supporting evidence remains limited by short follow-up periods and insufficient multicenter longitudinal datasets. At present, CPR can only be cautiously considered a tentative adjunct indicator to imply possible long-term metabolic risks rather than a definitive predictive tool; extended long-term cohort studies are urgently needed to verify these speculated associations and guide personalized lifelong follow-up plans for exposed offspring (51). Representative studies on CPR in obstetric complications are presented in Table 1. The mechanism underlying the role of CPR in obstetric complications is shown in Figure 2.

**TABLE 1:** Representative studies on CPR in FGR, PE, and HIP.

Authors	Population	Gestational weeks	CPR cutoff	Research type	Results	Conclusions
Nassr et al. (30)	Pooled cohorts from 1992–2014 multiple FGR studies	20–42 w	CPR < 1.08/below gestation-specific 5th percentile	Systematic review and meta-analysis	Abnormal CPR: 6–10-fold higher risk of emergency CS for fetal distress, 5-fold higher neonatal acidosis risk; Pooled sensitivity: fetal distress = 78.5%, 5-min Apgar <7 = 82.1%, NICU admission = 76.3%	CPR is an independent predictor of adverse perinatal outcomes (APO) in FGR; predictive performance superior to isolated UA/MCA Doppler
Kaya et al. (18)	120 singleton FGR fetuses followed to delivery	24–40 w	Below gestation-specific 5th percentile	Single-center prospective clinical cohort	APO incidence: 78.3% (abnormal CPR) vs. 21.7% (normal CPR), *P* < 0.001; Abnormal CPR independently predicts APO, OR = 6.82, 95%CI 3.21–14.50	Progressive CPR decline reflects exhausted fetal compensatory reserve, requiring intensified fetal surveillance and timely delivery
Hernandez-Andrade et al. (38)	2,986 low-risk singleton pregnancies	20–24 w	Below gestation-specific 5th percentile	Multi-center prospective cohort	Adjusted OR for subsequent preeclampsia = 5.8, 95%CI 2.6–13.0, *P* < 0.001; low CPR also predicts late-onset FGR and low birth weight	Second-trimester CPR acts as an early biomarker to predict preeclampsia and related fetal adverse outcomes
Ibrahim et al. (40)	128 women diagnosed with preeclampsia (tertiary hospital)	24–38 w	CPR ≤ 1.0	Cross-sectional clinical case series	Total abnormal CPR rate = 52.3%; abnormal CPR rate in severe PE = 86.7%; Adjusted prevalence ratio (APR) for severe PE = 5.0, 95%CI 1.28–29.14	The reduction degree of CPR is positively correlated with PE severity; early-onset PE has a higher proportion of abnormal CPR
Sangeereni et al. (49)	100 diabetic pregnancies (42 PGDM, 58 GDM singletons)	28–40 w	CPR < 1.3	Prospective clinical cohort	Mean CPR: PGDM 1.21 ± 0.32 vs. GDM 1.48 ± 0.41, *P* < 0.01; Abnormal CPR rate of PGDM = 38.1%; OR for composite APO = 4.23, 95%CI 1.89–9.45	Pregestational diabetes leads to more severe cerebroplacental hemodynamic imbalance; low CPR independently predicts neonatal adverse outcomes
Gabr et al. (46)	260 term singleton GDM pregnancies	37–40 w	Below gestation-specific 10th percentile	Cross-sectional clinical cohort	Neonatal APO incidence: 42.3% (abnormal CPR) vs. 10.5% (normal CPR); CPR predictive performance: sensitivity = 13.8%, specificity = 96.1%, overall accuracy = 86.9%	CPR presents high specificity but insufficient sensitivity when used alone; combined fetal biometry and amniotic fluid index are required for comprehensive risk evaluation

### 3.4 Application of CPR in other obstetric complications

#### 3.4.1 Preterm birth

Preterm birth (PTB) is defined as delivery occurring between 28 and 37 weeks of gestation, with a global incidence of 5–10%. It represents one of the leading causes of increased perinatal morbidity and mortality. Approximately 15 million preterm infants are born worldwide each year, among whom around one million die from preterm birth-related complications. The etiology of preterm birth is multifactorial, mainly associated with infection, excessive uterine distension, placental dysfunction, and maternal comorbidities. CPR is closely correlated with the onset, progression, and adverse perinatal outcomes of preterm birth, enabling objective quantification of fetal intrauterine hypoxia and hemodynamic compensatory remodeling. Placental insufficiency and uteroplacental circulatory disturbance constitute the key pathological basis of both spontaneous and iatrogenic preterm birth. Under such pathological conditions, compensatory dilatation of the fetal cerebral circulation and constriction of peripheral vessels occur, leading to an abnormally reduced CPR and indicating that the fetus is exposed to chronic intrauterine stress and hypoxia (5). In the clinical assessment of high-risk pregnancies, dynamic CPR monitoring allows accurate evaluation of fetal intrauterine tolerance and wellbeing, providing objective ultrasonographic evidence for delivery decision-making in women at high risk of preterm birth (52, 53). The presence of preterm labor signs combined with persistently abnormal CPR below the gestational age-specific cutoff value usually suggests decreased fetal intrauterine reserve. Continued expectant management may increase the risks of fetal distress, neonatal asphyxia, and long-term neurological impairment. Clinically, combined with gestational age, maternal comorbidities, uterine contraction status, and other hemodynamic parameters, CPR findings can serve as an important reference for individually weighing the benefits and risks between expectant tocolysis and pregnancy termination. This strategy facilitates rational timing of obstetric intervention, avoids unnecessary iatrogenic preterm birth, timely terminates adverse intrauterine conditions, and effectively improves the short- and long-term prognosis of preterm infants.

#### 3.4.2 Twin pregnancy

The incidence of twin pregnancy has increased annually with the development of assisted reproductive technology, representing a high-priority population in perinatal medicine. According to chorionicity, twin pregnancies are classified into MCDA and DCDA twins. Due to shared placental circulation, MCDA twins are prone to complications such as twin-to-twin transfusion syndrome (TTTS) and selective intrauterine growth restriction (sIUGR), carrying a higher risk of adverse perinatal outcomes than DCDA twins and singleton pregnancies. Twin pregnancies present a complex intrauterine developmental environment with distinctive blood flow distribution compared with singleton gestations, making accurate prenatal assessment essential. As a critical Doppler parameter reflecting the hemodynamic balance between fetal cerebral and placental circulation, CPR plays a prominent role in prenatal surveillance, complication prediction, and prognostic evaluation of twin pregnancy, and serves as a core reference indicator in perinatal management. Studies have demonstrated that CPR discordance between twins can effectively predict the risk of TTTS, sIUGR, and twin anemia–polycythemia sequence (TAPS) in complicated monochorionic twin pregnancies, and is closely correlated with intertwin weight discrepancy and adverse perinatal outcomes (54, 55). Meanwhile, abnormal CPR not only indicates chronic fetal intrauterine hypoxia and cerebral protective compensation, but also is significantly associated with an elevated risk of preterm birth. For preterm twin fetuses with reduced or abnormal CPR *in utero*, the postnatal risks of neurodevelopmental delay, impaired cognitive and motor function, and poor physical growth during infancy are markedly higher than in those with normal CPR. This suggests that CPR is a valuable biomarker for prenatal risk stratification and the evaluation of long-term neurodevelopmental and growth prognosis in twin pregnancies (56). Notably, this predictive utility is restricted to high-risk complicated twin cohorts, while cumulative evidence does not support universal routine CPR screening for all twin pregnancies, creating an important clinical distinction. A large retrospective cohort further confirms this limitation, Alexa Malika et al. performed an analysis of 247 twin pregnancies to explore the predictive value of CPR measured at 20–24 weeks of gestation for subsequent fetal growth restriction. The results showed that second-trimester CPR abnormalities were not significantly correlated with later onset of sIUGR, bilateral fetal growth restriction, or intertwin weight discrepancy exceeding 20%. In summary, CPR carries targeted clinical value for risk stratification and serial monitoring of complicated MCDA twins with confirmed sIUGR, TTTS or suspected TAPS, but cannot be recommended as a universal first-line screening tool for all twin pregnancies, especially in the second trimester. Standard twin surveillance should rely primarily on fetal growth trajectories, chorionicity grading, and umbilical artery Doppler; CPR is reserved as an adjunctive parameter for selective risk assessment in high-risk twin subgroups rather than routine universal testing (57).

#### 3.4.3 Intrauterine fetal demise

Intrauterine fetal demise (IUFD) represents one of the most severe adverse obstetric outcomes and involves a complex pathogenesis, with placental insufficiency and fetal intrauterine hypoxia serving as the primary predisposing factors. As a sensitive Doppler parameter reflecting fetal circulatory compensatory status, CPR is of great value in predicting IUFD. A systematic review by Catherine Elmes et al., incorporating nine studies with sample sizes ranging from 491 to 9772 pregnancies, demonstrated that CPR achieves moderate-to-high diagnostic accuracy for predicting fetal death, with a mean sensitivity of 79% and specificity of 78%, outperforming isolated measurement of MCA-PI or UA-PI (6). The predictive performance of CPR is even more prominent in SGA fetuses. A longitudinal study enrolling 941 SGA fetuses reported that the final CPR measurement yielded a predictive accuracy of 75.0% for IUFD, higher than 71.0% for UA-PI. Additionally, longitudinal monitoring of serial CPR changes did not significantly improve predictive efficiency, indicating that a single final CPR assessment can provide reliable evidence for IUFD risk stratification (32). At present, several clinical guidelines have incorporated CPR into the risk screening framework for IUFD. For pregnant women with abnormally reduced CPR, intensified prenatal surveillance, timely evaluation of placental function, and appropriate timing of pregnancy termination can effectively reduce the incidence of IUFD (52). Nevertheless, abnormal CPR is not a specific marker for IUFD. Comprehensive clinical judgment combined with other indicators, such as cardiotocography, biophysical profile score, and placental imaging examination, is required to avoid unnecessary overtreatment (10, 23).

## 4 Discussion and outlook

### 4.1 Conclusion

This review systematically summarizes existing domestic and international studies to comprehensively elaborate the physiological basis, ultrasonic detection specifications, and technical key points of CPR. It further systematically reviews its predictive value, clinical significance, and research progress in common high-risk obstetric complications, including fetal growth restriction, preeclampsia, hyperglycemia in pregnancy, preterm birth, twin pregnancy, and intrauterine fetal demise. As a sensitive ultrasonic indicator reflecting fetal cerebral blood flow redistribution and placental perfusion function, CPR can early identify fetal intrauterine hemodynamic abnormalities and placental insufficiency. It possesses important application value in the early screening of high-risk pregnancies, severity stratification, perinatal prognostic evaluation, and determination of clinical intervention timing. Moreover, CPR effectively compensates for the limitations in sensitivity and specificity of conventional monitoring methods such as isolated fetal growth parameters and umbilical artery blood flow, providing an important basis for earlier and more accurate assessment of fetal intrauterine status.

Although the CPR has been widely applied in obstetric ultrasound practice, its clinical popularization and standardized application still face multiple challenges. The cutoff criteria adopted across different studies remain inconsistent, and there are marked discrepancies in gestational age-specific reference ranges. The applicable scope and correction protocols for special populations, such as obese pregnancies, multiple gestations, and pregnancies conceived by assisted reproductive technology, remain to be further clarified. No standardized consensus has been established regarding the combined monitoring protocol integrating CPR with other hemodynamic parameters (e.g., uterine artery, ductus venosus, and middle cerebral artery) and serological biomarkers. In addition, some existing studies are limited by small sample sizes and short follow-up durations, resulting in insufficient evidence concerning long-term outcomes.

### 4.2 Future directions

Future research directions should focus on conducting large-scale, multicenter, prospective cohort studies to establish gestational age-specific normal reference ranges and unified diagnostic cutoff values for CPR applicable to different ethnicities and regions. It is necessary to optimize multi-index combined monitoring models and construct a comprehensive risk assessment system that balances sensitivity and specificity. Further exploration should be made into the associations between abnormal CPR and long-term health outcomes, including fetal neurocognitive development and childhood metabolic syndrome, so as to enrich evidence supporting its value in long-term prognostic evaluation. Meanwhile, promoting the application of intelligent ultrasound measurement and automatic interpretation technology can improve the standardization and reproducibility of CPR examination and reduce measurement errors.

With the accumulation of relevant evidence and ongoing clinical translation, standardized and precise application of CPR in the management of high-risk pregnancies is expected to be gradually realized. It can provide more reliable ultrasonic evidence for the dynamic monitoring of fetal intrauterine condition, the formulation of individualized diagnosis and treatment strategies, and the improvement of perinatal outcomes, thereby further promoting the development of perinatal medicine toward precision and individualization.
